# Chitinase 3-like 1 induces survival and proliferation of intestinal epithelial cells during chronic inflammation and colitis-associated cancer by regulating S100A9

**DOI:** 10.18632/oncotarget.5440

**Published:** 2015-09-28

**Authors:** Daren Low, Renuka Subramaniam, Li Lin, Tomoki Aomatsu, Atsushi Mizoguchi, Aylwin Ng, Arianna K. DeGruttola, Chun Geun Lee, Jack A. Elias, Akira Andoh, Mari Mino-Kenudson, Emiko Mizoguchi

**Affiliations:** ^1^ Gastrointestinal Unit, Department of Medicine, Massachusetts General Hospital and Harvard Medical School, Boston, MA, USA; ^2^ Laboratory of Cardiovascular Science, National Institutes on Aging, National Institutes of Health, Baltimore, MD, USA; ^3^ Division of Mucosal Immunology, Graduate School, Shiga University of Medical Science, Seta Tsukinowa, Otsu, Shiga, Japan; ^4^ Department of Immunology, Kurume University School of Medicine, Kurume, Fukuoka, Japan; ^5^ Department of Microbiology and Immunology, Warren Alpert School of Medicine, Brown University, Providence, RI, USA; ^6^ Department of Pathology & Cancer Center, Massachusetts General Hospital and Harvard Medical School, Boston, MA, USA; ^7^ Center for The Study of Inflammatory Bowel Disease, Massachusetts General Hospital and Harvard Medical School, Boston, MA, USA

**Keywords:** mammalian chitinase, colitis-associated cancer, bone marrow chimeras, RAGE, intestinal epithelial cells

## Abstract

Many host-factors are inducibly expressed during the development of inflammatory bowel disease (IBD), each having their unique properties, such as immune activation, bacterial clearance, and tissue repair/remodeling. Dysregulation/imbalance of these factors may have pathogenic effects that can contribute to colitis-associated cancer (CAC). Previous reports showed that IBD patients inducibly express colonic chitinase 3-like 1 (CHI3L1) that is further upregulated during CAC development. However, little is known about the direct pathogenic involvement of CHI3L1 *in vivo*. Here we demonstrate that CHI3L1 (aka Brp39) knockout (KO) mice treated with azoxymethane (AOM)/dextran sulphate sodium (DSS) developed severe colitis but lesser incidence of CAC as compared to that in wild-type (WT) mice. Highest CHI3L1 expression was found during the chronic phase of colitis, rather than the acute phase, and is essential to promote intestinal epithelial cell (IEC) proliferation *in vivo*. This CHI3L1-mediated cell proliferation/survival involves partial downregulation of the pro-apoptotic S100A9 protein that is highly expressed during the acute phase of colitis, by binding to the S100A9 receptor, RAGE (Receptor for Advanced Glycation End products). This interaction disrupts the S100A9-associated expression positive feedback loop during early immune activation, creating a CHI3L1^hi^ S100A9^low^ colonic environment, especially in the later phase of colitis, which promotes cell proliferation/survival of both normal IECs and tumor cells.

## INTRODUCTION

The causal relationship between inflammation and cancer is now widely recognized. In the case of inflammatory bowel disease (IBD), patients are predisposed to a 2% risk of developing colitis-associated cancer (CAC) by the 10^th^ year of ulcerative colitis (UC) diagnosis, with the risk increasing to 8% and 18% by 20 and 30 years respectively. The comparable risk of CAC has also been reported in longstanding Crohn's disease (CD) involving the colon, making CAC one of the major causes of death in IBD patients [1].

During the onset and/or flare of inflammation, innate immune activation provides the first response to counter pathogen exposure, releasing a milieu of molecules with anti-microbial and/or high reactive oxygen species (ROS) activities. Although these actions may be effective in promoting bacterial clearance, it also exerts high oxidative stress to intestinal epithelial cells (IECs) that may drive them into apoptosis and/or induce genomic mutation/instability. Hence, host IECs subsequently express pro-survival factors to counter effects of these harsh inflammatory events and promote epithelial restitution as part of recovery. However, promoting survival and proliferation of IECs that harbor genetic mutations induced as the consequence of the exaggerated inflammatory events will lead to tumor expansion. Since both inflammation and recovery can occur in the same region at the same time, a deeper understanding of how inducible specific factors promote intestinal homeostasis during the course of inflammation and how imbalance of these factors can exacerbate inflammation and/or tumorigenesis is needed.

Chitinase 3-like 1 (CHI3L1; aka Brp39) is a pseudo-chitinase that is usually undetectable in the colon of healthy individuals, but is expressed by IECs and macrophages in inflamed intestines [2]. CHI3L1 exhibits dual functions that promote Th2-type inflammation as well as enhance tissue remodeling and repair [3, 4]. During CAC development, a further increase in CHI3L1 expression was observed compared to healthy individuals, highlighting its potential role in tumorigenesis [5]. In many studies, CHI3L1 level was widely reported as a biomarker for many solid tumors [6]. Previous *in vitro* studies have suggested that CHI3L1 promotes proliferation in colon cancer cell lines and increases xenografted tumor load and angiogenesis [7]. However, the direct *in vivo* role of CHI3L1 during tumorigenesis, in particularly in an inflammation setting, has not been explored.

Like CHI3L1, the damage-associated molecular (DAMP) protein S100A9 is also usually undetectable in healthy colons, but can be induced in colonic mucosa during colitis [8]. Intracellular S100A9 can associate with NADPH complex to regulate the ROS pathway and oxidative burst whereas extracellular S100A9 possesses antimicrobial activity through sequestration of zinc metal to a level that is lower than what is needed by most microbes [9–11]. During inflammation, secreted S100A9 can bind onto the cell surface Receptor for Advance Glycation End product (RAGE) and activate NF-kB signaling that binds back onto the S100A9 promoter creating a transcription positive feedback loop [8]. In this way, inflammatory immune responses can be augmented through the continuously activated NF-kB signaling pathway. However, high levels of endogenous S100A9 can lead to oxidative stress-induced apoptosis, as shown by overexpression or high-dose stimulation of S100A9 in several cancer cells including colon cancer [12, 13]. These observations suggest a presence of a regulatory factor to regulate the negative effects of S100A9 on host cells under inflammatory conditions.

Despite the fact that the expressions of both CHI3L1 and S100A9 are reportedly induced during inflammation, possible cross-regulation of these two proteins has not been explored. This hypothesis is based on previous reported genome-wide gene expression profiling that showed an upregulation of S100A9 in *Mycobacterium tuberculosis*-infected CHI3L1 (Brp39) knockout (KO) mice, suggesting a potential cross-regulating mechanism that creates an inverse relationship between CHI3L1 and S100A9 expression [14]. In this study, we demonstrate that CHI3L1 expression in IECs can provide a protection against chronic colitis by promoting the survival and proliferation of IECs both *in vitro* and *in vivo*. These anti-apoptotic and pro-proliferative effects of CHI3L1 are achieved though the downregulation of S100A9, by binding/blocking the RAGE that cuts the S100A9-RAGE positive feedback loop, resulting in a pro-proliferative CHI3L1^hi^ S100A9^low^ environment. This leads to the survival and proliferation of not only normal IECs under inflammatory conditions that facilitate the improvement of colitis, but also potential cancer progenitor cells that may promote CAC.

## RESULTS

### CHI3L1 expression in IECs is critical for epithelial survival and proliferation during chronic intestinal inflammation

**Figure 1:**
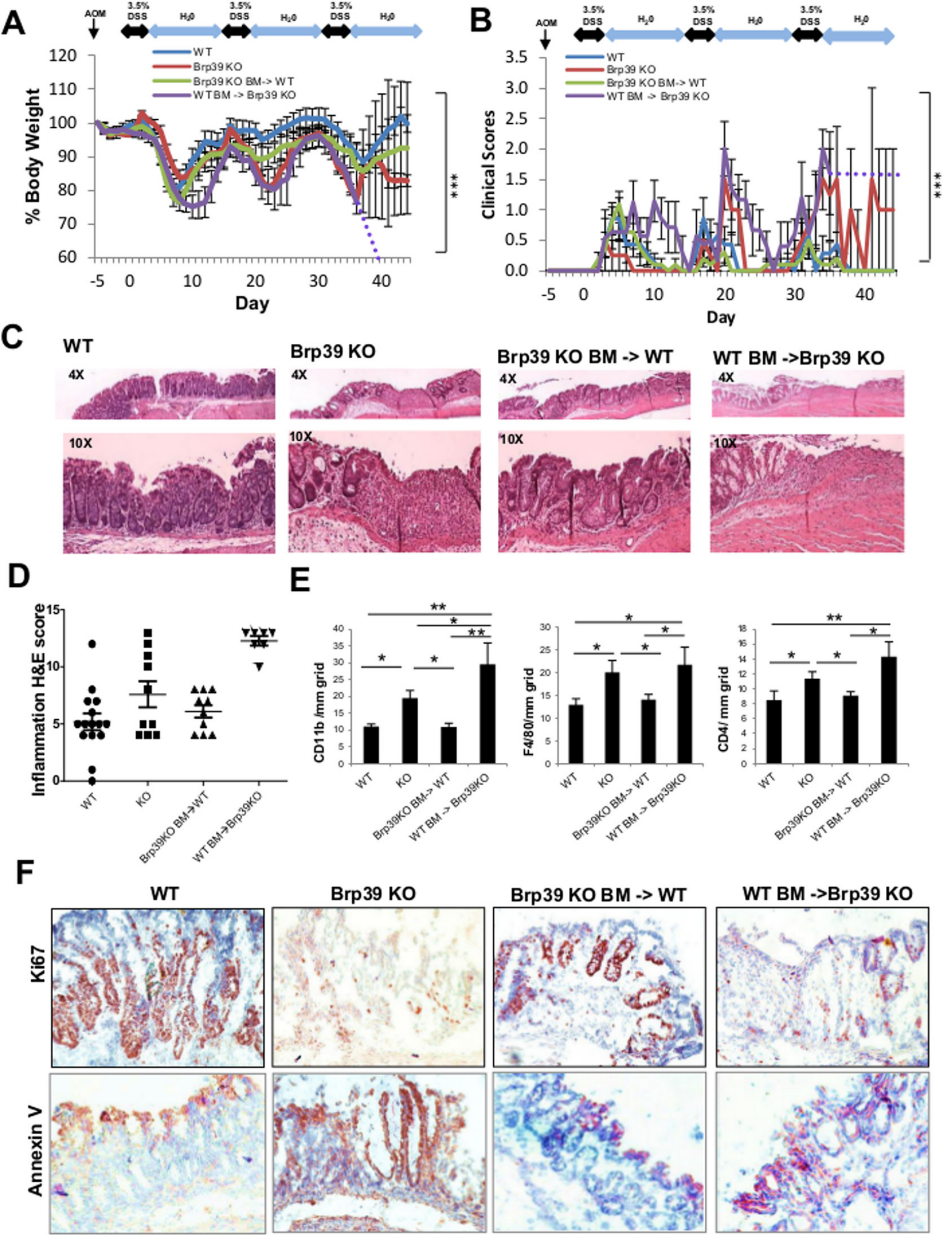
Brp39 KO mice developed severe colitis but can be partially ameliorated with IECs CHI3L1 expression and exacerbated with hematopoietic cells CHI3L1 expression **A, B.** Body weight and clinical scores of AOM/DSS treated mice were recorded daily. WT BM-> Brp39 KO mice was prematurely sacrificed at day 36 after their body weight fell below the level that was approved by the animal protocol (75% of the original body weight); subsequent body weight and clinical scores of this group were extrapolated and shown by dotted lines. **C, D.** Colonic H&E sections of AOM/DSS treated mice are blindly scored by certified pathologist. **E.** Frozen colonic sections were stained with CD11b, F4/80 and CD4 anti-bodies and positive cells were quantified per mm^2^ grid. **F.** IHC staining of colonic sections using Ki67 and Annexin V anti-bodies of the AOM/DSS treated mice is shown. Objective, 10 ×. *p < 0.05, **p < 0.01, ***p < 0.001.

To characterize the phenotypic role of CHI31L expression induced during chronic colitis and tumorigenesis, WT and Brp39 KO mice were given a single intraperitoneal injection of a carcinogen AOM and 3.5% DSS in the drinking water for five days and normal water for the subsequent 10 days for 3 cycles (AOM/DSS) (Supplementary Figure 1A). Brp39 KO mice showed delayed body-weight (BW) loss during the first cycle of DSS treatment (acute), but subsequently lost significantly more BW on the second and the third cycles of DSS (chronic phase) as compared to WT mice (Figure 1A). In addition, Brp39 KO mice exhibited much more severe clinical symptoms as judged by diarrhea, bloody stool and hunching posture, especially during the second and the third cycles of DSS treatments (Figure 1B), suggesting that the lack of CHI3L1 contributes to the exacerbation of chronic colitis.

To further investigate how CHI3L1 deficiency exacerbates chronic colitis, H&E sections of the mouse colon were examined microscopically. The Brp39 KO mice that had undergone AOM/DSS treatment showed partial mucosal erosions and atrophy, whereas much less epithelial damage was observed in the WT mice (Figure 1C, 1D). In addition, significant inflammatory responses with infiltration of CD11b+, F4/80+ and CD4+ immune cells in the lamina propria were observed in the colon of the Brp39 KO mice compared to that of WT mice (Figure 1C, 1E). These results suggest that the CHI3L1 expression in the intestines induced during the intense inflammatory conditions can provide the IECs with a certain degree of protection. Indeed, a distinctly higher degree of Ki-67 positive proliferative cells were detected in AOM/DSS treated WT mice, as compared to those of Brp39 KO mice(Figure 1F). Conversely, the Brp39 KO mice colonic section showed more intense staining of the apoptotic marker Annexin V, providing further evidence that CHI3L1 expression is required for the survival and proliferation of IECs (Figure 1F). In the tumor area, CHI3L1 expression on IEC was highly upregulated, which promotes cell proliferation (Ki67 positive staining) and suppresses cell death (Annexin V negative staining) (Supplementary Figure 1B).

To delineate the roles of CHI3L1 expression in hematopoietic versus non-hematopoietic cells, we generated two models of bone marrow transplanted (BMT) chimeric mice: Brp39 KO BM-transplanted WT (Brp39 KO BM -> WT) mice and WT BM-transplanted Brp39 KO (WT BM-> Brp39 KO) mice. AOM/DSS treatment in the Brp39 KO BM -> WT BMT-chimeric mice resulted in a BW profile that is intermediate between WT and Brp39 KO mice, suggesting that the CHI3L1 expression in the non-hematopoietic cells, presumably IECs, can partially ameliorate colitis (Figure 1A). Conversely, WT BM-> Brp39 KO BMT-chimeric mice showed delayed recovery after the first DSS cycle and subsequently began to dramatically lose their BW in the third DSS cycle (Figure 1A). In addition, they frequently showed severe clinical symptoms (Figure 1B) and developed lethal colitis as indicated by a high mortality (Supplementary Figure 2A, 2B). These observations suggest that the CHI3L1 expression in the hematopoietic cells mediates and augments immunologic responses during chronic DSS treatment. Colonic microscopic findings also confirmed the high inflammatory profile with diffuse mucosal erosion in the WT BM-> Brp39 KO mice (Figure 1C, 1D, 1E). Conversely, the Brp39 KO BM-> WT mice displayed a histological profile that is intermediate between WT and Brp39 KO mice (Figure 1C, 1D, 1E), suggesting again that the CHI3L1 expression in the non-hematopoietic cells, in particular in IECs, partially protect mice from colitis exacerbation, presumably through IEC survival and restitution. Indeed, the Brp39 KO BM-> WT BMT chimeric mice displayed much more increased numbers of Ki-67 positive IECs, and smaller numbers of Annexin V positive cells as compared to those of WT BM-> Brp39 KO mice (Figure 1F). Taken together, these findings suggest two functionally distinct roles of CHI3L1 in non-hematopoietic cells versus hematopoietic cells. The former may promote IECs survival and proliferation and the latter may activate immunological response.

### CHI3L1 expression in IECs promotes tumor growth and expansion

**Figure 2:**
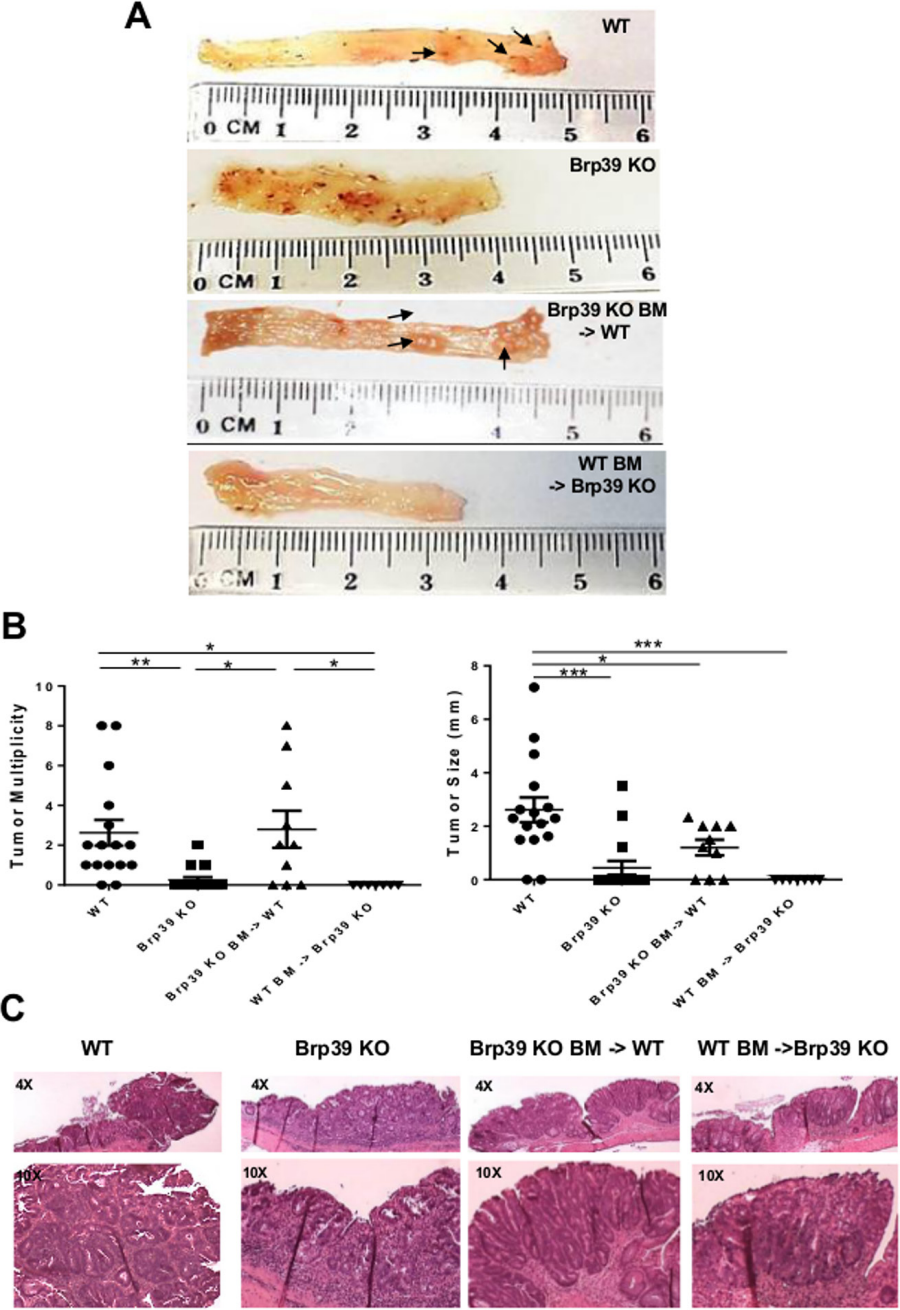
CHI3L1 expression on IECs significantly promotes tumorigenic changes in distal part of colon **A.** Whole colon tissues from AOM/DSS-treated mice are shown (left: proximal, right: distal). **B.** Macroscopic tumor multiplicities and size were counted and measured. **C.** Tumor/dysplasia region of H&E colonic section from AOM/DSS-treated mice are shown. *p < 0.05, **p < 0.01, ***p < 0.001.

To explore the role of inducible CHI3L1 in CAC, WT- and Brp39 KO-AOM/DSS-treated mice was examined for tumor formation. Polypoid lesions were detected in the 88% of the AOM/DSS-treated WT mice, predominately in the distal part of the colon. In contrast, only 16% of AOM/DSS-treated Brp39 KO mice showed grossly detectable polypoid lesions (Figure 2A). Notably, both the number and size of tumors were significantly larger in the WT mice than in the Brp39 KO mice (Figure 2B). Colonic histology revealed increased dysplastic foci in many of the polypoid lesions in the AOM/DSS-treated WT mice as compared to those of the Brp39 KO mice (Figure 2C). The role of hematopoietic versus non-hematopoietic cell-derived CHI3L1 in tumor formation was next examined using the AOM/DSS-treated BM-chimeric mice (Brp39 KO BM-> WT and WT BM -> Brp39 KO). Polypoid lesions can be macroscopically determined in the Brp39 KO BM-> WT mice, but not observed in the WT BM-> Brp39 KO mice (Figure 2A, 2B). Together, these results indicate that the proliferative and pro-survival role of CHI3L1 particularly in IECs can promote tumor growth/expansion upon neoplastic transformation.

### Fecal CHI3L1 levels serve as one of the informative biomarkers for detecting CAC progression during chronic colitis

**Figure 3:**
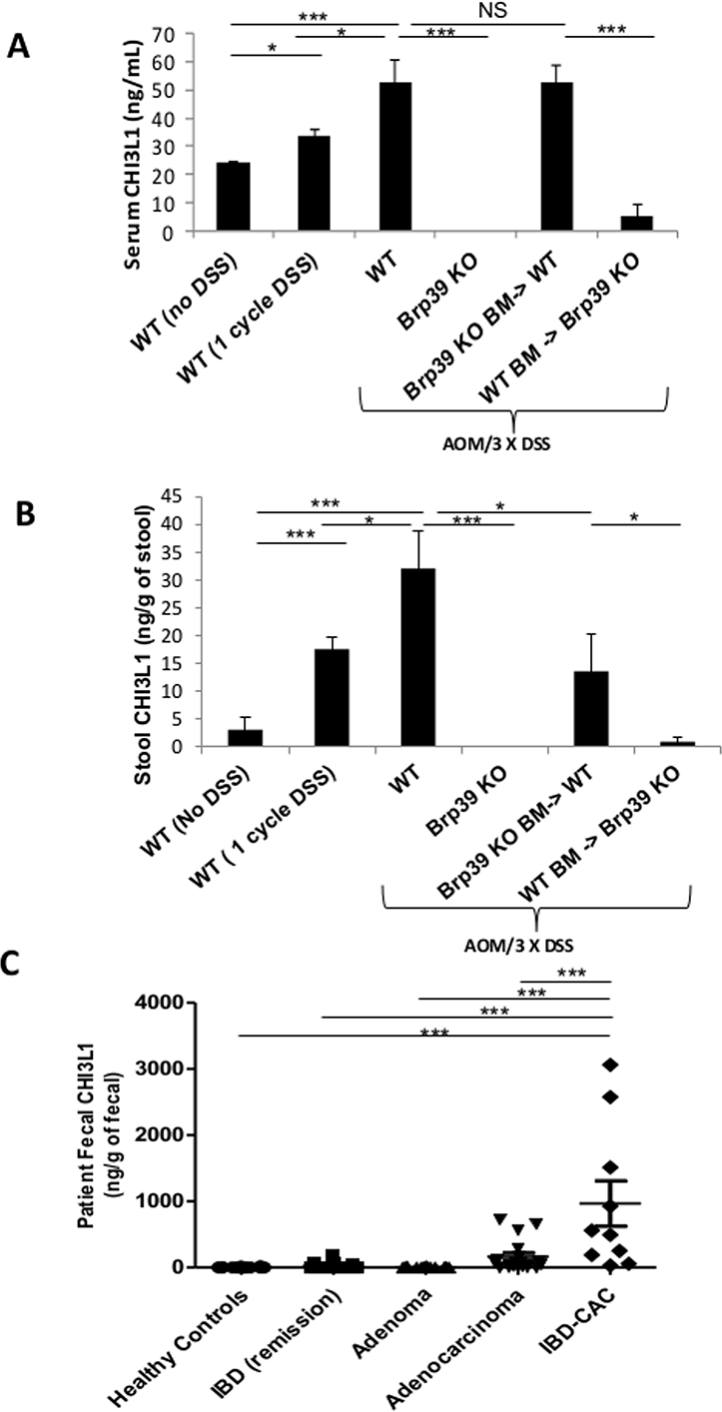
Stool CHI3L1 levels as non-invasive as well as sensitive biomarker for CAC development **A, B.** Mice serum and stool were collected and quantified for CHI3L1 levels using ELISA. **C.** CHI3L1 levels from human stool samples were quantified using ELISA. *p < 0.05, **p < 0.01, ***p < 0.001, NS, not significant.

Since colonic CHI3L1 is known to be inducibly expressed in IECs during the course of IBD which is further overexpressed when CAC develops, quantifying CHI3L1 levels using easily accessible sample collection and methods may help to develop a new diagnostic strategy for predicting the neoplastic changes of IECs in IBD patients. Therefore, serum and stool samples were collected from untreated WT mice, WT mice that were given 1 cycle of DSS for 5 days (i.e. acute colitis), and WT mice that were given AOM/DSS treatment (i.e. chronic colitis and CAC). ELISA quantification showed a step-wise increase in CHI3L1 levels in both serum and stool of the untreated WT mice to WT mice that had undergone 1 cycle of DSS and to AOM/DSS treated WT mice, indicating that CHI3L1 levels are positively associated with the progression of colitis and CAC (Figure 3A, 3B). In addition, the AOM/DSS-treated Brp39 KO BM-> WT mice, but not WT BM-> Brp39 KO mice, showed high levels of CHI3L1 in both serum and stool samples (Figure 3A, 3B). The result indicates that the CHI3L1 in these samples mainly originated from a non-hematopoietic source, presumably IECs. In addition, in untreated WT mice, the fecal CHI3L1 levels are much lower compared to the serum CHI3L1 levels in this experiment (Figure 3A, 3B). Furthermore, based on the Pearson correlation analysis, CHI3L1 stool level versus tumor multiplicity/size showed positive association in AOM/DSS-treated WT mice (Supplementary Figure 2C). These results were recapitulated in human fecal samples. Healthy individuals showed almost undetectable levels of fecal CHI3L1, whereas a step-wise increment of fecal CHI3L1 was detected in IBD patients, which were then significantly upregulated upon CAC development in the colon (Figure 3C). In addition, fecal CHI3L1 levels were much higher in CAC patients compared to other sporadic colorectal cancer patients (Figure 3C). The background of patients in this study is summarized in Table 1. In this study, IBD with CAC as well as IBD patients have similar levels of inflammation and selected inflammatory parameters including white blood cell counts, CRP, and serum albumin as shown in Table 2. Taken together, these results provide a proof of concept that fecal CHI3L1 levels reflect disease progression and may serve as one of the most accurate and non-invasive biomarkers for the detection of neoplastic changes in IBD patients.

**Table 1:** Demographic characteristics of all patients in this study

	Normal	IBD	Adenoma	AdenoCa	CAC
Number	20	19	22	18	12
Age (mean ± SD)	56.9 ± 16.7	29.5 ± 18.1	69.1 ± 7.4	67.1 ± 9.4	52.9 ± 10.9
Sex (Male/Female)	11/9	12/7	18/4	10/8	9/3
Race (Asian)	20	19	22	18	12
Year of disease duration (median)	–	7.6	–	–	21.5
Disease location					
UC (pancolitis/left-sided/protocolitis)	–	7/3/2	–	–	6/3/1
CD (ileal/ileocolonic)	–	2/5	–	–	0/2
Colitis-associated cancer histology	–	–	–	–	0/1/1
Adenocarcinoma	–	–	–	18	8
Mucinous carcinoma	–	–	–	–	2

AdenoCa: sporadic adenocarcinoma, CD: Crohn's disease; LGD, low grade dysplasia; HGD, high grade dysplasia; UC, ulcerative colitis

**Table 2:** Inflammatory parameters of IBD and CAC patients in this study

Parameters (mean ± SD)	IBD	CAC	*T*-test (*p*-value)
WBC (/μl)	5377 ± 1122	5842 ± 1563	0.34
CRP (mg/dl)	0.16 ± 0.28	0.29 ± 0.26	0.21
Serum Albumin (g/dL)	4.4 ± 0.3	4.2 ± 0.6	0.12

CAC: colitis-associated carcinoma; CRP: C-reactive protein; IBD; inflammatory bowel disease; WBC: white blood cells

### CHI3L1 and S100A9 expressions are inversely related

Since CHI3L1 and S100A9 molecules are inducibly expressed in the intestine during IBD, but Brp39 KO mouse was previously reported to show an up-regulation of S100A9 expression during inflammation, we therefore examined the potential association between these two molecules [15]. To do this, human colonic SW480 cells were transfected with pcDNA4 CHI3L1-expression plasmid. Interestingly, CHI3L1 overexpression resulted in a reduction of S100A9 expression level in these cells (Supplementary Figure 3A). However, in the presence of pan-chitinase inhibitor, S100A9 expression was restored in the CHI3L1-overexpressing cells. In addition, siRNA-mediated knockdown of CHI3L1 resulted in an increase of S100A9 expression in SW480 cells (Supplementary Figure 3B). These findings suggest that CHI3L1 exerts a negative regulatory effect on S100A9 expression in IECs.

### CHI3L1 is a competitive inhibitor of S100A9 binding to RAGE

**Figure 4:**
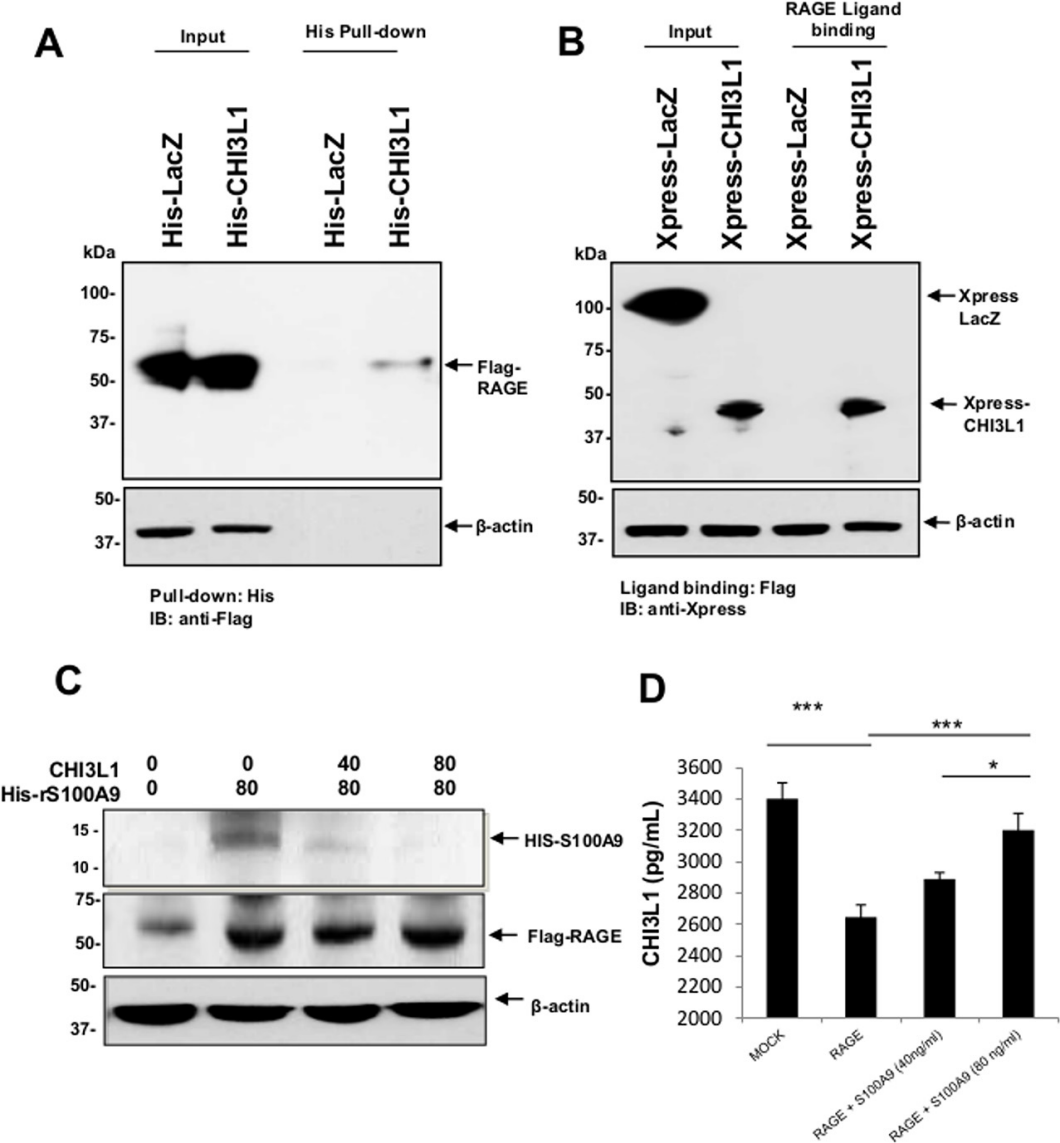
Extracellular (secreted) CHI3L1 binds to the cell-surface associated RAGE competitively with S100A9 **A, B.** Total protein cell lysate from His-Xpress-tagged CHI3L1 or LacZ (control) overexpressed SW480 cells were incubated with cells overexpressing Flag-tagged RAGE and cross-linked. Subsequently, proteins were pulled down using nickel affinity beads or anti-Flag bead and immunoblotted using anti-Flag or anti-Xpress. **C.** His-tagged rS100A9 was added into RAGE-overexpressed SW480 cells in the presence or absence of different concentration of purified CHI3L1, cross-linked, pulled down using nickel affinity beads, separated using SDS-PAGE and immunoblotted with anti-His antibody. **D.** SW480 IECs were transfected with mock or RAGE overexpressing plasmids and 24 hours later, 0, 40 or 80 ng/ml of rS100A9 was added into the culture medium for additional 24 hours. Culture supernatant was collected and quantified for soluble CHI3L1 concentration using ELISA as shown in the Materials and Method section. *p < 0.05, ***p < 0.001.

One of the major pathways that induce the endogenous expression of S100A9 is through binding of the RAGE receptor with its specific ligands, including AGEs and S100A9 itself. Thus, we hypothesize that CHI3L1 may interact with RAGE during inflammation and ultimately prevents the RAGE/RAGE-ligand interaction for the activation of S100A9 expression. To test this hypothesis, a ligand-binding assay was first performed by incubating total lysate from SW480 cells overexpressing His/Xpress-tagged CHI3L1 or LacZ (control) protein with overexpressed Flag-tagged RAGE protein. Subsequently, pulldown of His-tagged CHI3L1 protein, but not His-tagged LacZ control protein, with His-nickel beads co-precipitated Flag-tagged RAGE (Figure 4A) showed the RAGE-CHI3L1 interaction. In addition, ligand binding assay of Flag-tagged RAGE with anti-Xpress co-precipitation of CHI3L1 (Figure 4B) further confirmed the RAGE-CHI3L1 binding.

Since both CHI3L1 and S100A9 expressions are induced during inflammation and both bind to their common receptor RAGE, we next hypothesized that CHI3L1 and S100A9 compete for the binding to RAGE. To test this hypothesis, a constant concentration of His-tagged rS100A9 protein was incubated with SW480 cells, which were transfected with pCDNA3.1 RAGE-expression plasmid, in the presence of various concentrations of purified CHI3L1 protein. Subsequently RAGE ligand binding assay and immunoblotting using anti-His antibody showed decreasing amounts of detectable His-tagged S100A9 that were bound with the RAGE in the presence of increasingly higher concentrations of CHI3L1 protein (Figure 4C). In contrast, the amount of free detectable CHI3L1 in the cell culture supernatant decreased when SW480 cells were transfected with pCDNA3.1 RAGE plasmid but was restituted, in a dose dependent manner, when exogenous rS100A9 protein was introduced in the culture medium (Figure 4D). Taken together, these results indicate that both CHI3L1 and S100A9 can bind to the RAGE competitively depending upon the concentration gradient between the two proteins. Thus binding of CHI3L1 to RAGE disrupts the S100A9-RAGE-induced positive feedback loop of S100A9.

### CHI3L1 binding to RAGE enhances STAT3-, β-catenin- and NF-κB-associated signaling pathways

**Figure 5:**
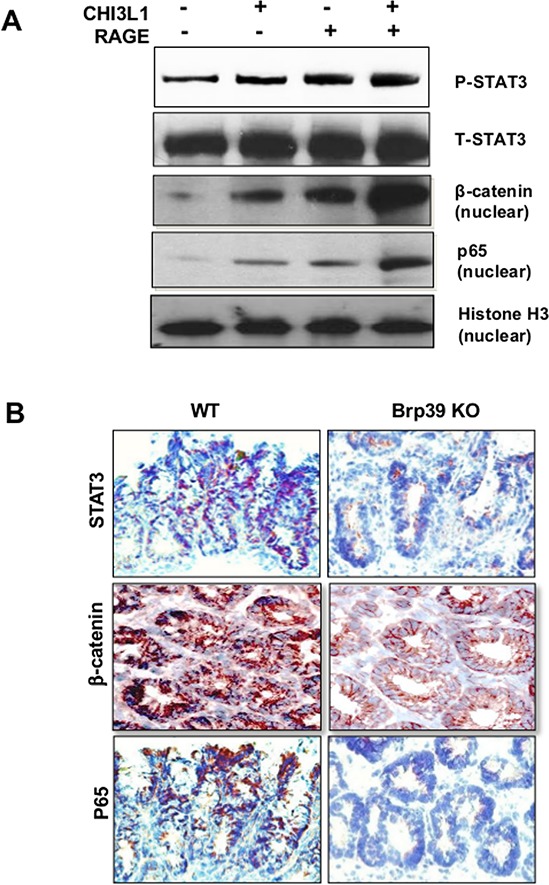
CHI3L1-RAGE ligation synergistically activates STAT3, β-catenin and p65 **A.** SW480 IECs were transfected with CHI3L1 and/or RAGE expressing plasmid and total or nuclear protein lysate was resolved by SDS-PAGE and immunoblotted with anti-phospho/total STAT3, anti-β-catenin or anti-p65 antibodies. **B.** Frozen colonic sections of AOM/DSS treated WT and Brp39 KO mice were IHC stained with anti-STAT3, anti-β-catenin or anti-p65 antibodies. Objective, 20 ×.

Previous *in vitro* studies demonstrated that STAT3, β-catenin, NF-κB and AKT are the four main signaling pathways activated by CHI3L1 in IECs [5, 16, 17]. Therefore, the status of these signaling pathways upon the ligation of CHI3L1 and RAGE was next determined. SW480 cells were mono-transfected with either pcDNA4 CHI3L1- or pcDNA3.1 RAGE-expression plasmid that showed increased amounts of STAT3-, β-catenin- and NF-κB-activation (Figure 5A). The activation of these signaling pathways were further enhanced when these cells were co-transfected with the CHI3L1- and the RAGE-expression plasmids (Figure 5A). However, the co-transfection did not affect AKT activation (data not shown). *In vivo*, colonic sections from AOM/DSS treated WT mice clearly displayed strong nuclear localization of STAT3, β-catenin as well as NF-κB, whereas Brp39 KO mice predominately exhibited a cytoplasmic staining pattern of these transcriptional factors (Figure 5B and Supplementary Figure 4).

### Stoichiometry and balancing reactions between CHI3L1 and S100A9 control the fate of cell death or survival in a RAGE-dependent manner

**Figure 6:**
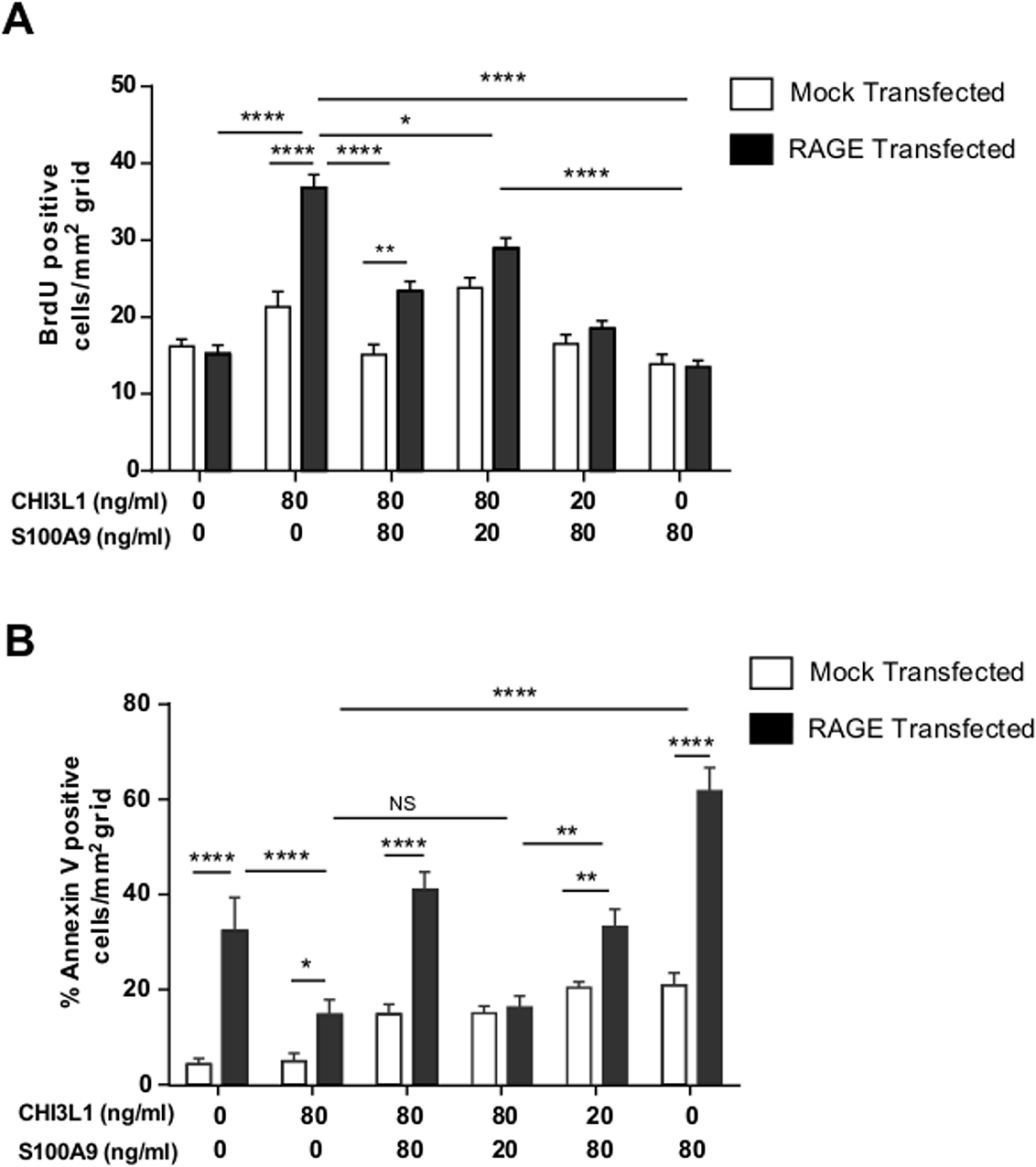
Highest IECs proliferation under CHI3L1^hi^S100A9^low^ stimulation while CHI3L1^low^S100A9^hi^ promotes apoptosis in a RAGE-dependent manner **A, B.** SW480 cells were transfected with mock or RAGE expressing plasmid and stimulated with rCHI3L1 and/or rS100A9 at different concentration and pulsed with BrdU for 1 hour. Cells were stained with anti-BrdU or anti-Annexin V antibodies and positive cells were quantified per mm^2^ grid. *p < 0.05, **p < 0.001, ****p < 0.0001, NS, not significant.

To determine how the stoichiometrical balance between CHI3L1 and S100A9 can impact cell fate under intestinal inflammation, pCDNA3.1 RAGE- or -mock expression plasmid was transfected to SW480 cells and co-stimulated with different concentrations of CHI3L1 and S100A9 proteins and subsequently stained with anti-BrdU antibody to evaluate the extent of proliferation and anti-Annexin V antibody for the extent of apoptosis upon treatment. A BrdU-incorporation rate was significantly increased in the presence of CHI3L1^hi^ S100A9^low^ stimulation and this effect was greatly enhanced with the RAGE-overexpression on the cells (Figure 6A). This co-stimulatory condition also yielded the lowest rate of apoptotic cells (Figure 6B). Conversely, CHI3L1^low^ S100A9^hi^ co-stimulatory condition resulted in the highest rate of apoptotic cell death as determined by Annexin V staining (Figure 6B), and RAGE overexpression significantly enhanced the apoptotic cell death. These observations suggest that the concentration-dependent balance between CHI3L1 and S100A9 induced during intestinal inflammation drives IECs to either proliferation or apoptosis.

### Temporal co-expression of CHI3L1 and S100A9 from acute to chronic colitis correlates with cell proliferation and apoptosis *in vivo*

**Figure 7:**
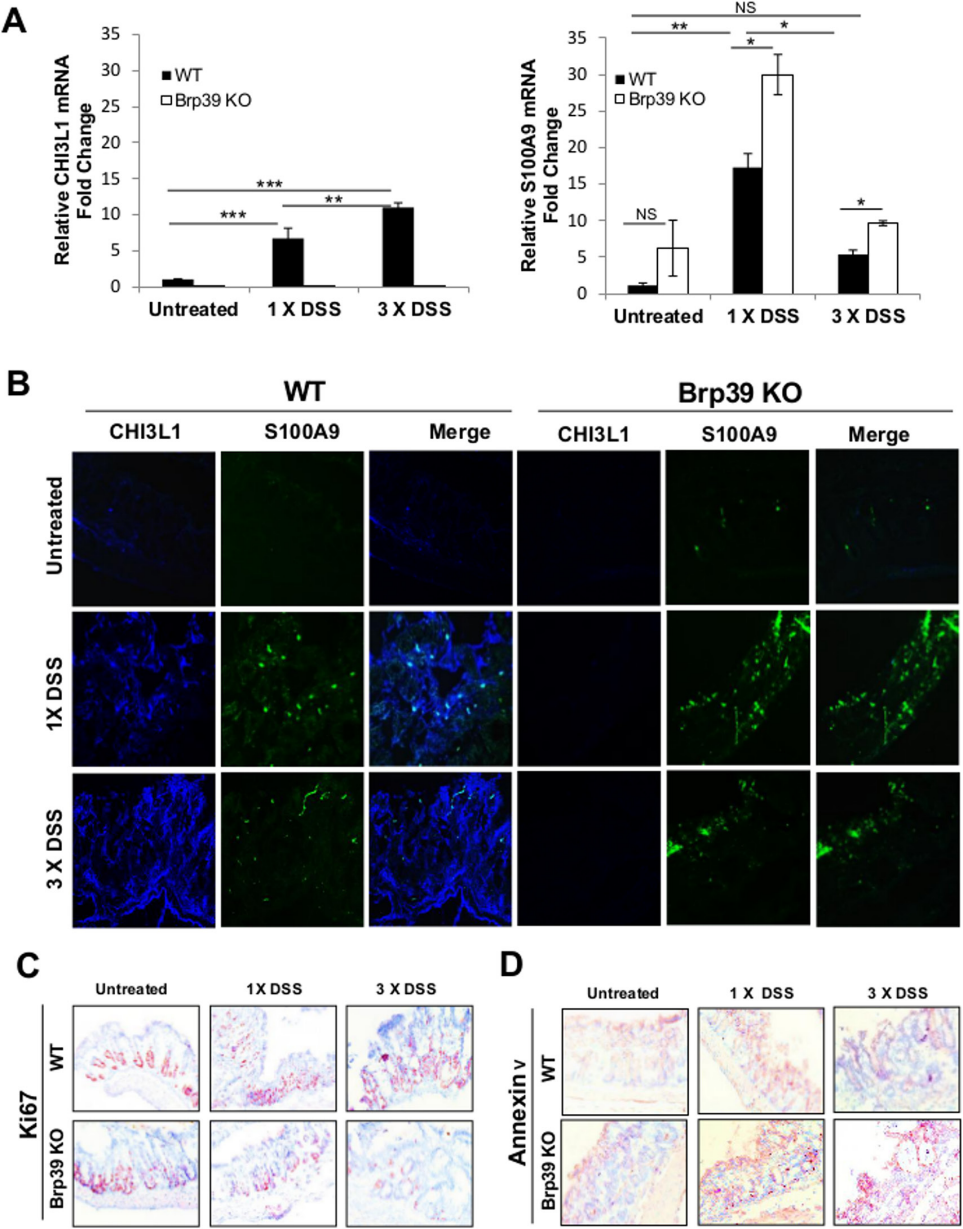
Co-expression levels progression of CHI3L1 and S100A9 from steady state to acute and chronic colitis phase correlates with colonic proliferation and apoptosis **A.** Whole colon mRNA from respective mice group were quantified for CHI3L1 and S100A9 expression using real-time PCR. **B.** Frozen colonic sections were co-stained with rabbit anti-CHI3L1 and rat anti-S100A9 primary antibodies and subsequently incubated with Alexa-fluor 647-conjugated anti-rabbit and FITC-conjugated rabbit-anti-rat secondary antibodies and visualized under a confocal microscope. **C, D.** IHC was performed on colonic sections using anti-Ki67 or anti-Annexin V antibodies. Objective, 10x. *p < 0.05, **p < 0.01, ***p < 0.001, NS, not significant.

To understand the co-expression pattern of CHI3L1 and S100A9 during the progression from acute to chronic colitis *in vivo* and what effects the extent of this induction have on colonic homeostasis, WT and CHI3L1 KO were either treated with 1 cycle of DSS for 5 days (acute colitis), or AOM/DSS (chronic colitis/CAC). Subsequently, Quantitative PCR and immunofluorescence microscopic analysis were performed to quantify and visualize the colonic expression levels of CHI3L1 and S100A9. During the onset of DSS-induced acute colitis, CHI3L1 expression is induced in WT mice that further up-regulated during the chronic phase (Figure 7A). In contrast, S100A9 expression was highly induced during the course of acute DSS-induced colitis in WT mice, but dropped significantly during the chronic phase of colitis (Figure 7B). While CHI3L1 expression is undetectable in the Brp39 KO mice in any treated groups, low level of S100A9 expression can already be detected at steady state in these mice that significantly up-regulated during onset of acute DSS-induced colitis in a manner that is much higher than those of WT, which then subsequently decreased during the chronic phase of colitis. However, the S100A9 level in Brp39KO mice was significantly higher as compared to WT during the chronic phase of colitis (Figure 7A, 7B). These results suggest that S100A9 may act as an intensive/prompt responder during the acute phase of DSS-induced colitis, which then decreased during the course of chronic colitis as a result of the increased levels of competitive soluble CHI3L1.

Finally, the expression patterns of CHI3L1 and S100A9 were next examined in terms of the degree of cell death and cell survival during the course of intestinal inflammation. In the WT mice, colonic IHC staining revealed a high Ki-67 labeling index during the onset of acute DSS-induced colitis, which further expanded as it progressed into the chronic phase, showing a strong association with the step-wise increase of CHI3L1 expression during the distinct disease phases (Figure 7C). However in Brp39 KO mice, Ki-67 positive cells dramatically decreased during the acute phase of colitis and almost undetectable during the chronic phase, indicating the CHI3L1 expression is crucial for the proliferation of IECs during the course of intestinal inflammation (Figure 7C). Conversely, in the Brp39 KO mice, Annexin V levels were increased during the transitional phases in the course of colitis, presumably a result of imbalance between high S100A9 and no CHI3L1 expression levels (Figure 7C, 7D). Taken together, these results reflect that the temporal and spatial stoichiometry of CHI3L1 and S100A9 may regulate the final fate of IECs in terms of cell death and cell survival during the course of chronic inflammation and inflammation-associated tumorigenesis in the large intestine.

## DISCUSSION

Increased CHI3L1 expression appears to be an important hallmark of inflammation and cancer and has been frequently observed in patients with poor prognosis. Here, we show that CHI3L1 expression is required for IECs survival by promoting the proliferation of these cells during the transitional phases of colitis and CAC, in part through the competitive inhibition of S100A9 binding to the RAGE.

Temporal expression levels of a gene during different stages and severity of disease can provide informative insights about gene functions. CHI3L1 expression levels appear to be highest during the recovery phase from acute colitis, which reflects one of its major functions in tissue restitution by promoting cell survival and proliferation. Lack of CHI3L1 can result in delayed recovery from acute inflammation, but excessively increased expression levels of CHI3L1 can also be associated with pathogenic conditions involving hyper proliferation. For instance, patients with idiopathic pulmonary fibrosis (IPF) showed significant CHI3L1 expression in the lungs, but when these patients experienced acute exacerbations, CHI3L1 expression dropped to a level between those of IPF patients and the control group [18]. In this current study, the induction phase of acute colitis renders a lower level of CHI3L1 expression compared to the later chronic phase of colitis. These results suggest that CHI3L1 might not be the first inflammation-responsive factor, but may be important during the recovery phase by promoting tissue restitution and remodeling. Interestingly the expression of S100A9 appears to be inversely correlated with that of CHI3L1. Our observations are consistent with a previous report that showed S100A9 is an early responder expressed during the initial stage of acute colitis within the first two weeks of initiation but is absent during the chronic phase of colitis [19]. Since RAGE is a pattern recognition receptor, RAGE and RAGE-ligand interactions mostly activate common proinflammatory signaling pathways. We found both CHI3L1- and S100A9-RAGE interactions stimulated significant phosphorylation of MAPK p42/p44 and p38 and weak phosphorylation of JNK, STAT3, and caspase 3 p17 (data not shown.). In addition, our *in vivo* and *in vitro* results demonstrated that expression of CHI3L1 and S100A9 are inversely correlated in acute- and chronic-DSS-induced colitis in WT and Brp39 KO mice. Therefore, it is likely that negative regulation and outcome of S100A9-RAGE signaling axis by CHI3L1 is concentration dependent and not through a distinct inflammatory signaling, which we tested above. Furthermore, previous studies have shown that low concentration of S100A9 induces pro-tumor proliferative effects [11]. High concentration (>80 ng/ml) of CHI3L1 in CAC environment seems to favorably bind to RAGE, which interaction enhances epithelial proliferation and tumorigenesis, and therefore the effect of S100A9-RAGE may be negligible because of the competitive binding. However, it is still plausible that the other signaling pathways could contribute partially to negative regulation of S100A9 in a concentration-dependent or -independent manner.

Furthermore, functions of CHI3L1 and S100A9 in cancer cells are also inversely correlated each other. For instance, S100A9 inhibits human cervical cancer cell metastasis [20]. Gastric patients with high counts of S100A9 positive cells also associate with less lymph node metastasis [21]. Suppressing S100A9 using inhibitor of DNA binding (1d1) promotes the migratory and invasive potentials of breast cancer. In contrast, many reports have confirmed the tumor promoting and pro-metastatic functions of CHI3L1 in solid tumors [22].

When induced, the biological roles of CHI3L1 toward enteric microbes are distinct from those of S100A9. Overexpression of S100A9 downregulates the invasive abilities of *Listeria* and *Salmonella* into epithelial cells exerted through its antimicrobial properties [23]. However, overexpression of CHI3L1 promotes the adhesion/invasion of potentially pathogenic bacteria into IECs [2, 24]. In particular, enteric microbes can penetrate into tumor cells and release growth promoting products that may facilitate colonic tumor progression [25]. All these observations clearly support the notion that although both CHI3L1 and S100A9 are induced during the course of inflammation and tumorigenesis, they are likely to exhibit a cross-regulatory pattern, in this case presumably by binding competitively to the common RAGE during a disease state.

Although S100A9 is pro-apoptotic at high concentration, it is still required for CAC development since S100A9 KO mice showed fewer incidences of AOM/DSS-induced CAC [26]. High amount of S100A9 is induced during the initial phase of inflammation, which promotes chemotaxic potentials of immune cells and exerts anti-microbial and ROS activities that, in turn, may induce high oxidative stress to IECs. The oxidative stress can compromise host cell genomic stability and induce mutations that may lead to carcinogenic changes of IECs. Following the expression of S100A9, CHI3L1 expression is upregulated in IECs in the later phase. CHI3L1 binds to the RAGE receptor and cuts the S100A9 positive feedback loop, leading to the downregulated expression of S100A9. CHI3L1, with proliferative effects, then promotes survival and expansion of both normal and tumor cells. Indeed, low concentration of S100A9 has been reported to have pro-tumor proliferative effects. During systemic inflammation, serum levels of S100A9 were reported to be in the range of 1–6 mg/mL [27]. However in cancer patients, S100A9 serum levels were reported to be significantly lower in nano- to μg/ml range, despite being higher than those of healthy controls [28]. These results clearly indicate that a shift of S100A9 concentration from high to low is required for the neoplastic progression of IECs during the course of chronic colitis. In contrast, there are a couple of previous publications, which suggest that S100A9 directly regulates the tumor developments as well as metastasis in human colorectal cancer patients [29, 30]. Although the biological half-life of S100A9 is unclear at this moment, only free S100A9 protein, which does not bind to RAGE, will be detected in serum levels, and therefore the increased serum levels of S100A9 may be a result of increased CHI3L1 binding to RAGE. Here we demonstrate that the dynamic process of the neoplastic progression of IECs in chronic colitis is regulated by the increased production of CHI3L1, which reciprocally regulates the expression of S100A9 under inflammatory conditions. However we still cannot completely deny a possibility that the drop in S100A9 is just a consequence of tissue destruction/tumor progression and can decrease irrespective of CHI3L1.

In summary, this study presents the essential role of CHI3L1 in promoting CAC by enhancing survival and proliferation of IECs and neoplastic cells through the downregulation of S100A9 and its effects during the preceding inflammation. We also show that the analysis of CHI3L1 levels in non-invasively collected feces serves as a convenient and reliable modality to predict the neoplastic changes during the course of chronic colitis.

## MATERIALS AND METHODS

### Animals and ethics statement

CHI3L1 KO mice on an inbred C57Bl/6 background were obtained from Drs. Anthony Coyle and Alexander Kozhick (MedImmune LLC, Gaithersburg, MD), originally generated by Drs. Chun Geun Lee and Jack A. Elias (Brown University Warren Alpert School of Medicine, Providence, RI) and housed in the Massachusetts General Hospital specific pathogen-free facility, under an Institutional Animal Care and Use Committee approved protocol [15].

### Tumor induction

CAC was induced in 8–10 week old Brp39 KO and C57Bl/6 wild-type (WT) mice by single injection of azoxymethane (AOM; Sigma-Aldrich, St. Louis, MO) at 12 mg/kg. Five days later, mice were treated with 3.5% dextran sulfate sodium (DSS) for 5 days and replaced with normal water for the next 10 days for 3 cycles. Body weight and clinical symptoms (diarrhea, blood in stool and hunching posture; 0 or 1) were monitored daily. Mice were sacrificed on day 45 and gross macroscopic tumors were counted and measured under an Olympus SZ-PT dissection microscope (Olympus, Central Valley, PA). H&E sections of the colons were blindly scored by a pathologist with expertise in GI pathology based on previously reported criteria [31].

### Protein-protein interaction assay

4 × 10^5^ SW480 cells were seeded on 6-well plate and transfected with 2.5 μg of His-Xpress tagged pCDNA4-CHI3L1- or -LacZ-, or 2.5 μg of Flag tagged pCDNA3.1-RAGE-expression plasmid, which was a gift from Dr. Lin Li (National Institutes on Aging, National Institutes of Health, Baltimore, MD) [2, 32]. Ligand binding assay was performed by adding the total protein lysate of His-Xpress tagged pCDNA4-CHI3L1- or -LacZ transfected SW480 cells to Flag-RAGE expressing SW480 cells. Binding assay was performed at 4°C in the presence of DTSSP crosslinker (Life Technologies, Grand Island, NY). Subsequently, membrane-associated protein was extracted and His-Select Nickel Affinity Gel (Sigma Aldrich) was used to purify CHI3L1 bound RAGE. CHI3L1-RAGE interaction was detected by SDS-PAGE and immunoblotting with anti-Flag or anti-Xpress antibody [32].

### Immunohistochemical analysis

Colonic frozen sections (4 μm thick) were stained with the respective primary antibodies using the avidin-biotin-complex system (Vector laboratories, Burlingame, CA). The following primary antibodies were used for staining: anti-Ki-67 and anti-β-catenin (Abcam, Cambridge, MA, USA), anti-Annexin V (Thermo Scientific, Rockford, IL, USA), as well as anti-NF-κB p65 and anti-STAT3 (Santa Cruz Biotechnology, Dallas, Texas, USA) antibodies.

### Enzyme-linked immunosorbent assay (ELISA)

Mouse CHI3L1 levels in serum and stool (resuspended 10 mg/ml in PBS) were detected using ELISA kit purchased from R&D systems (Minneapolis, MN, USA) and performed according to the manufacturer's instructions. Supernatant of SW480 cells that were transfected with pcDNA3.1-RAGE and stimulated with different concentrations of recombinant S100A9 (Life technologies) were collected and detected with human CHI3L1 using the same ELISA kit (R&D systems). Optical density was measured at 450 nm using Auto-Reader Model 680 (Bio-rad, Hercules, CA). Human stool was collected from patients with IBD, colonic adenoma, sporadic colon cancer, or CAC at the Division of Gastroenterology and Division of Mucosal Immunology, Shiga University of Medical Science (Otsu, Japan) from April 2007 to November 2009. Similarly, 10 samples from healthy individuals were obtained at the same University. One gram of fresh stool was collected and stored at −80°C until the stool CHI3L1 levels were measured by ELISA kit (R&D systems) as previously described [33].

### Bone marrow transplantation (BMT)

Bone marrow cells were aseptically isolated from the femurs and tibia of WT and Brp39 KO mice as previously described [34]. Recipient mice were irradiated with 6 Gray from a ^137^Cs Gamma Cell source and intraperitoneally injected with 10^7^ donor bone marrow cells. Four weeks later, mice were subjected to AOM/DSS treatment as described above.

### Western blotting

Total proteins from RAGE and/or CHI3L1 expression plasmid transfected cells were extracted using 0.1% NP-40 lysis buffer as previously described [35]. Nuclear proteins were extracted, incubating cells with hypotonic buffer (20 mM Tris-HCl pH 7,4, 10 mM NaCl, 3 mM MgCl_2_) for 15 minutes on ice and centrifuge at 3000 rpm for 10 min at 4°C. The pellet was then lysed with 1% NP-40 lysis buffer for 30 minutes on ice and supernatants were collected by centrifuging at 13,000 rpm for 30 minutes [35]. SDS-PAGE and Western blot were performed as previously described using anti-phospho/total STAT3 (Cell Signaling, Danvers, MA), anti-β-catenin (Abcam), anti-Histone H3 (Cell Signaling), and anti-p65 (Santa Cruz, Dallas, Tx) primary antibodies [36].

### Quantitative PCR

Colonic total RNA was extracted using Trizol reagent (Life Technologies, Grand Island, NY) according to the manufacturer's instructions. One microgram of RNA was reverse transcribed using iScript cDNA synthesis kit (Bio-rad). PCR reaction was set up using the iQTM SYBR Green Supermix (Bio-rad) and amplified the following primer pairs: human CHI3L1 (F: 5′-GAAGACTCTCTTGTCTGTCGGA, R: 5′-AATGGCGGTACTGACTTGATG), mouse CHI3L1 (F: 5′-TCCTGATGCTGCTCCAGAG, R: 5′-TATGCATGTTGTCGCTGCTG), human S100A9 (F: 5′-GTCATAGAACACATCATGGAG, R: 5′-GGCCTGGCTTATGGTGGTG), mouse S100A9 (F: 5′-CCAACAAAGCACCTTCTCAG, R: 5′-GCTGATTGTCCTGGTTTGTG).

### Cell proliferation and apoptosis analysis

SW480 cells were transfected with pcDNA3.1-RAGE or control expression plasmid for 48 hours, stimulated with different concentrations of purified-CHI3L1 protein (Quidel, San Diego, CA) and/or rS100A9 (Life technologies) for the next 24 hours and then pulsed with 10 μmol/L 5-bromo-2-deoxyuridine (BrdU; Sigma Aldrich) in cell culture medium for 1 hour. Cells were then trypsinised and cytocentrifuged using Shandon Southern Cytocentrifuge (Cheshire, England). Samples were then fixed in 100% acetone for 10 minutes and stained using anti-BrdU (Serotec, Oxford, England) or Annexin V (Thermo scientific) antibody.

### Confocal microscopy

Mouse colonic cryosections were fixed in acetone for 10 minutes and air-dried at room temperature, blocked in 5% goat and horse serum for 30 minutes at room temperature and then co-stained with rabbit-anti-CHI3L1 (Affinity Bioreagent, Golden, CO) and rat-anti-S100A9 (Abcam) antibodies at 4°C overnight. Sections were washed and stained with Alexa Fluor 647 goat-anti-rabbit IgG (Invitrogen) and FITC horse-anti-rat IgG (Vector Laboratories) secondary antibodies for 30 minutes at RT. The sections were washed with PBS, mounted in Vecta-shield (Vector) and analyzed under the Bio-Rad Radiance 2000 confocal microscope.

### Statistical analysis

Student's *T*-test or One-Way and Two-Way ANOVA with post-hoc Tukey Test, when appropriate, was performed using the Graphpad Prism Software for multiple comparison experiments. Values were presented in mean ± SEM. *P* ≤ 0.05 was considered as statistically significant.

## SUPPLEMENTARY DATA MATERIALS AND METHODS FIGURES


