# Supplementary material for: Chitinase 3-like 1 induces survival and proliferation of intestinal epithelial cells during chronic inflammation and colitis-associated cancer by regulating S100A9
Source: Oncotarget. 2015 Sep 28;6(34):36535–50. doi: 10.18632/oncotarget.5440 (PMC4742194; doi:10.18632/oncotarget.5440)
Supplement: Supplementary file 1 [file oncotarget-06-36535-s001.pdf]

SUPPLEMENTARY DATA

MATERIALS AND METHODS

Tumor induction

CAC was induced in 8-10 week old Brp39 KO and C57Bl/6 wild-type (WT) mice by single injection of azoxymethane (AOM; Sigma-Aldrich, St. Louis, MO) at 12 mg/kg. Five days later, mice were treated with 3.5% dextran sulfate sodium (DSS) for 5 days and replaced with normal water for the next 10 days for 3 cycles. Body weight and clinical symptoms (diarrhea, blood in stool and hunching posture; 0 or 1) were monitored daily. Mice were sacrificed on day 45 and gross macroscopic tumors were counted and measured under an Olympus SZ-PT dissection microscope (Olympus, Central Valley, PA).

Immunohistochemical analysis

Colonic paraffin-embedded sections (5 μm thick) or frozen sections (4 um thick) were stained with the respective primary antibodies using the avidin-biotin-complex system (Vector laboratories, Burlingame, CA). The following primary antibodies were used for staining: anti-Ki-67, anti-Annexin V (Thermo Scientific, Rockford, IL, USA), anti-RAGE (MyBioSource, San Diego, CA), anti-CHI3L1 (Affinity Bioreagent, Golden, CO), anti-β-catenin, or anti-STAT3 (Santa Cruz Biotechnology, Dallas, Texas, USA) antibodies. Control normal rabbit IgG and rat IgG were purchased from Vector.

IEC line preparation and quantitative PCR

4×10^5 SW480 human IECs were seeded on 12-well plate and transfected with 0.5 μg of His-Xpress tagged pCDNA4-CHI3L1 or -LacZ plasmid in the presence or absence of the 2.5 mM pan-chitinase inhibitor (caffeine) for 48 hours. Colonic total RNA was extracted using Trizol reagent (Life Technologies, Grand Island, NY) according to the manufacturer's instructions. One microgram of RNA was reverse transcribed using iScript cDNA synthesis kit (Bio-rad). PCR reaction was set up using the iQTM SYBR Green Supermix (Bio-rad) and amplified the following primer pairs: mouse CHI3L1 (F: 5′-TCCTGATGCTGCTCCAGAG, R: 5′-TATGCATGTTGTCGCTGCTG), mouse S100A9 (F: 5′-CCAACAAAGCACCTTCTCAG, R: 5′-GCTGATTGTCCTGGTTTGTG).

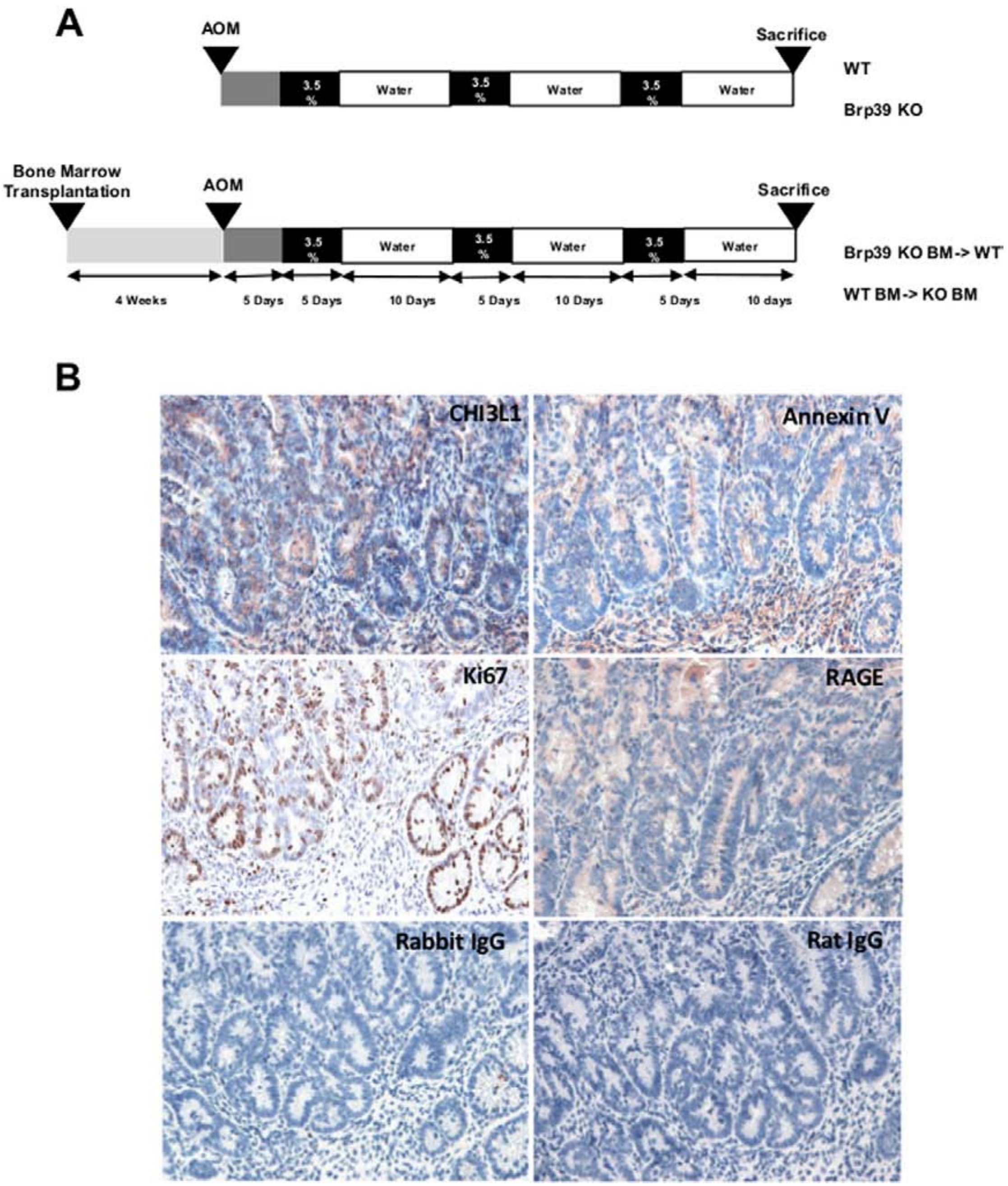

Supplementary Figure S1: Schematic representation of the *in vivo* AOM/DSS treatment, and increased CHI3L1 expression in the dysplastic IECs obtained from AOM/DSS-treated WT mice. A. The AOM/DSS treatment was performed on WT and Brp39 KO, as well as bone marrow transplanted chimera mice. **B.** Paraffin-embedded colonic sections of AOM/DSS treated WT mice were stained with anti-CHI3L1, RAGE, Ki67, or Annexin V antibodies with immunohistochemical standard technique. Objective, 20 ×.

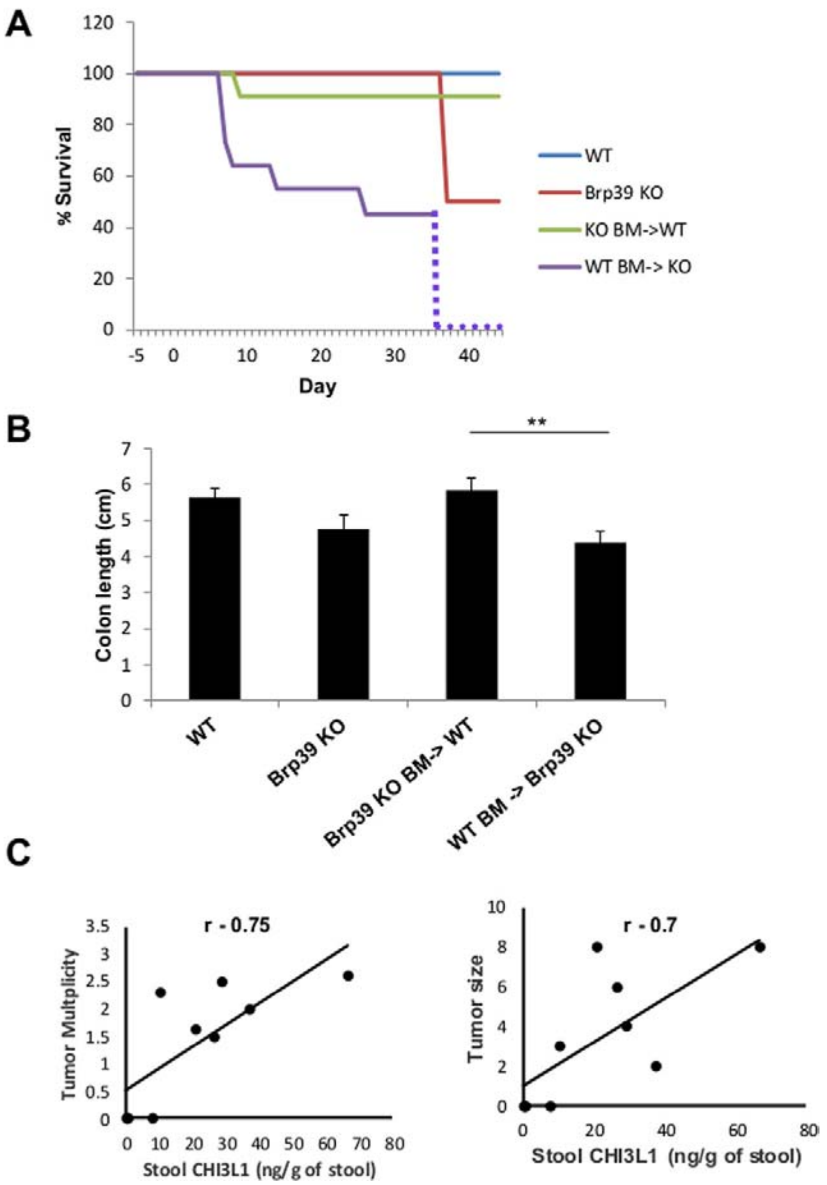

Supplementary Figure S2: Survival rate, colonic length and tumor/stool CHI3L1 association in the AOM/DSS treated mice. A. Percentage of mice that survive during AOM/DSS treatment is shown. WT BM-> Brp39 KO BM chimera mice were prematurely sacrificed on day 36 after their body weight dropped below the level that was approved by the animal protocol (75% of the original body weight). **B.** Colonic length of the AOM/DSS treated mice was measured at the treatment end point. **C.** A Pearson correlation analysis was performed to analyze stool CHI3L1 level versus tumor multiplicity as well as tumor size in WT mice with AOM/DSS treatment. r represents the pearson correlation coefficient index. $**p < 0.01$.

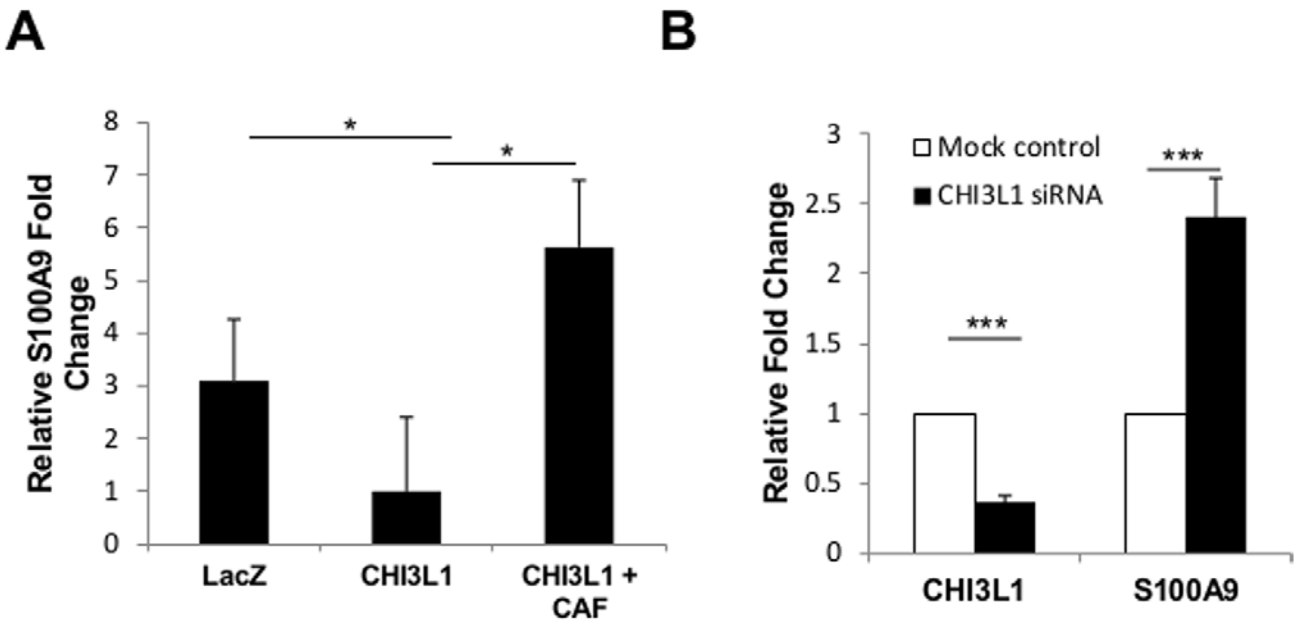

Supplementary Figure S3: Over-expression or knockdown of CHI3L1 results in down- or up-regulation of S100A9, respectively, *in vitro*. A. SW480 IECs were transiently transfected with LacZ- (control) or CHI3L1-expressing plasmid in the presence or absence of the 2.5 mM pan-chitinase inhibitor (caffeine) for 48 hours and the mRNA level of S100A9 was subsequently determined using real-time PCR. **B.** SW480 cells were transfected with CHI3L1 siRNA for 48 hours and mRNA levels of CHI3L1 and S100A9 were quantified using real-time PCR. *$p < 0.05$, ***$p < 0.001$.

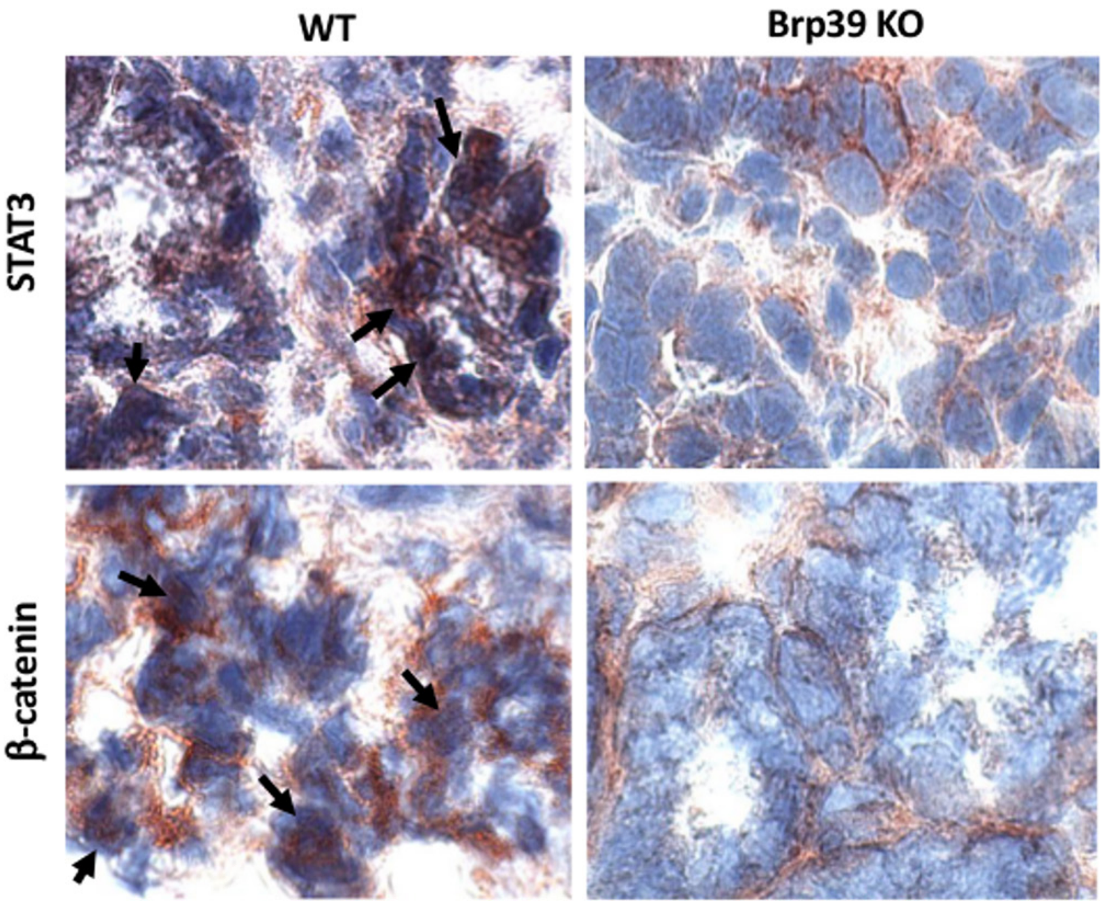

Supplementary Figure S4: Nucleic translocation of STAT3 and β-catenin in the colons from WT mice but not CHI3L1 KO mice with AOM/DSS treatment. Frozen colonic sections of AOM/DSS treated WT and Brp39 KO mice were IHC stained with anti-STAT3 or anti-β-catenin antibodies. Arrows show the nucleic staining pattern. 100 × oil objective.
